# Supplementary material for: The interactome of intact mitochondria by cross-linking mass spectrometry provides evidence for coexisting respiratory supercomplexes
Source: Mol Cell Proteomics. 2017 Dec 8;17(2):216–32. doi: 10.1074/mcp.RA117.000470 (PMC5795388; doi:10.1074/mcp.RA117.000470)
Supplement: Supplemental Data [file supp_RA117.000470_133922_0_supp_23978_fzffwf.zip › spectra_annotation/mito_spectra_annotation/72-1-3-1-6-1.pdf]

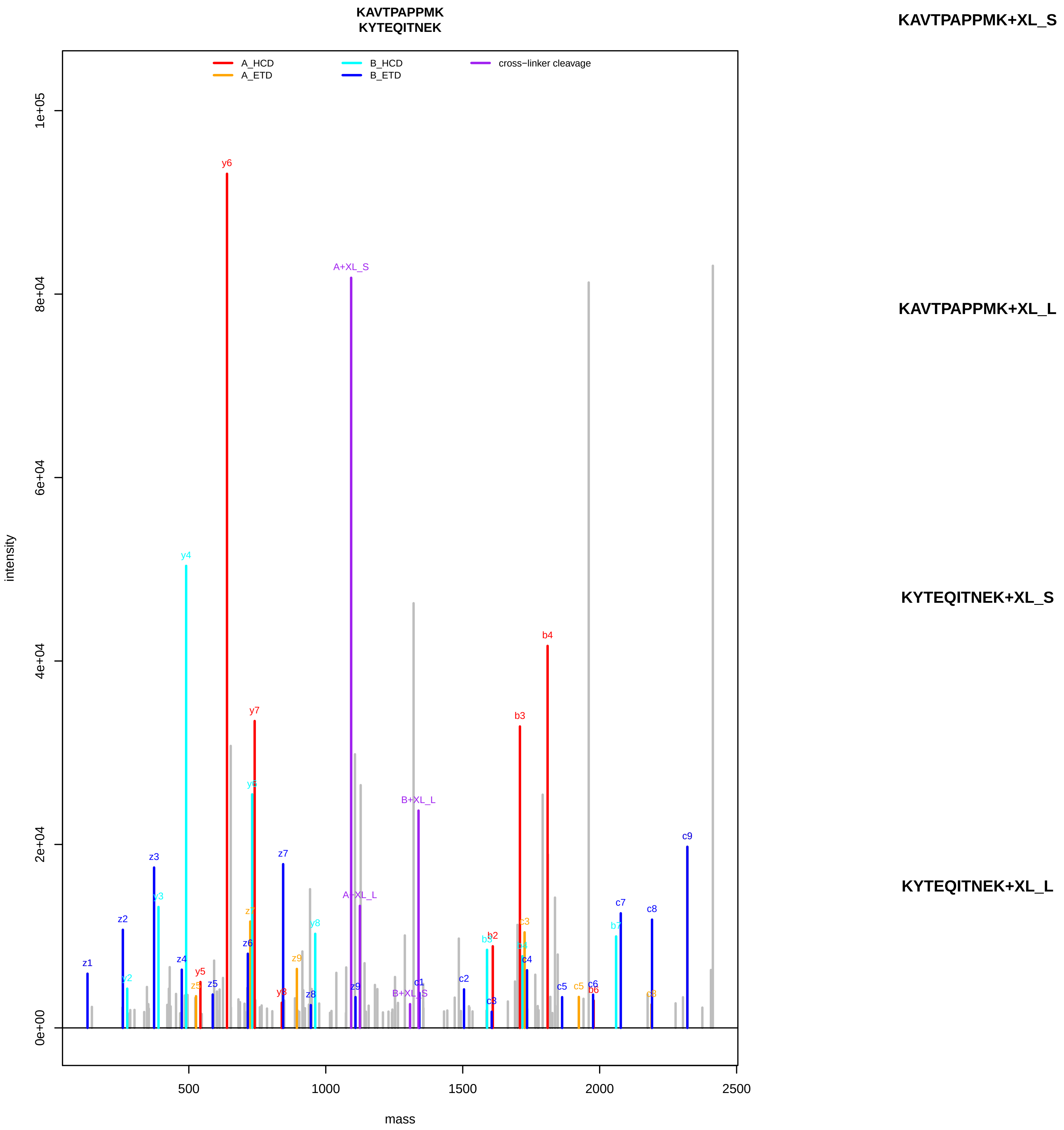
KAVTPAPPMK
KYTEQITNEK
KAVTPAPPMK+XL_S
KAVTPAPPMK+XL_L
KYTEQITNEK+XL_S
KYTEQITNEK+XL_L
A_HCD
A_ETD
B_HCD
B_ETD
cross−linker cleavage
intensity
mass
0e+00
2e+04
4e+04
6e+04
8e+04
1e+05
500
1000
1500
2000
2500
y6
A+XL_S
y4
b4
y7
b3
B+XL_L
A+XL_L
c9
z3
z7
y3
c7
c8
z2
y8
c3
b2
b5
b7
z6
z9
z1
z4
y5
z5
c2
c4
c5
c6
b6
c1
B+XL_S
z8
z9
c3
